# Coexistence of kidney and lung hydatid cyst in a child: A case report

**DOI:** 10.1016/j.rmcr.2024.102138

**Published:** 2024-11-28

**Authors:** Seyed Hossein Mirlohi, Sanaz Tajfirooz, Hojatollah Raji, Sima Akhavan

**Affiliations:** a Pediatric Respiratory and Sleep Medicine Research Center,Children's Medical Center,Tehran University of Medical Sciences, Tehran, Iran; b Children's Medical Center, Pediatric Center of Excellence, Tehran, Iran; c Department of Pediatric Surgery, Tehran University of Medical Sciences, Tehran, Iran; d Tehran University of Medical Sciences, Tehran, Iran

## Abstract

Hydatid cyst (HC) is a zoonotic disease that often affects regions where animal husbandry is common and preventive measures are not taken. This disease mostly affects the liver and the lungs. Involvement of other organs, such as the kidney, musculoskeletal system, and intracranial structures, is rare. In this case report we will be discussing a patient who was diagnosed with bacterial pulmonary empyema without proper response to treatment. In further management, a ruptured hydatid cyst was diagnosed along with a renal hydatid cyst.

## 1 Introduction

Hydatid cyst (HC) is a zoonotic condition commonly found in areas where livestock farming is prevalent and preventative measures are not taken. The disease is caused by the cestode Echinococcus granulosus [1], in which humans acquire by ingesting the eggs of a parasite. This condition is endemic in parts of the Mediterranean, South America, the Middle East, Australia, and South Africa [2]. In addition, hydatid cysts are also endemic in Russia, Central Asia, New Zealand and the rest of Africa. In adults, this disease mostly affects the liver, with infection rates between 60% and 80 %, followed by the lungs, which are affected in 20%–30 % of cases [1,3]. The disease also affects the musculoskeletal system, kidneys, and brain [3]. However in pediatrics, Echinococcus often spreads predominantly in the lungs. Thus, 60 % of lung infections occur in the right lung, particularly in the lower lobes, affected in 50%–60 % of cases [4,5]. In patients from endemic areas presenting with cystic lesions, HC should be considered as the primary diagnosis. However, identifying HC can be challenging both clinically and through imaging in cases of complex cysts. Alternative diagnoses that may be considered include pulmonary abscess, bronchogenic cyst, primary lung sarcoma lung metastases, hematoma, mesothelioma and granuloma [[6], [7], [8]].

## 2 Case presentation

**Fig. 1:**
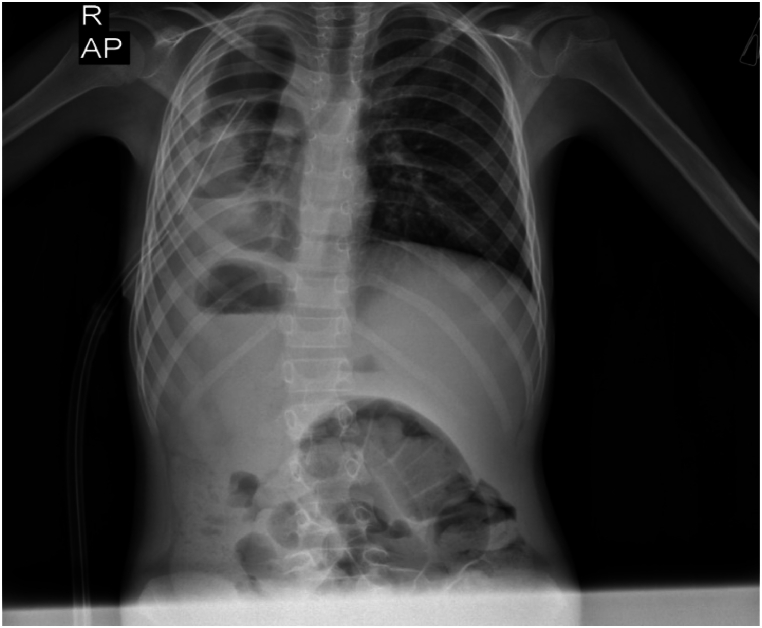
First chest X-ray.

**Fig. 2:**
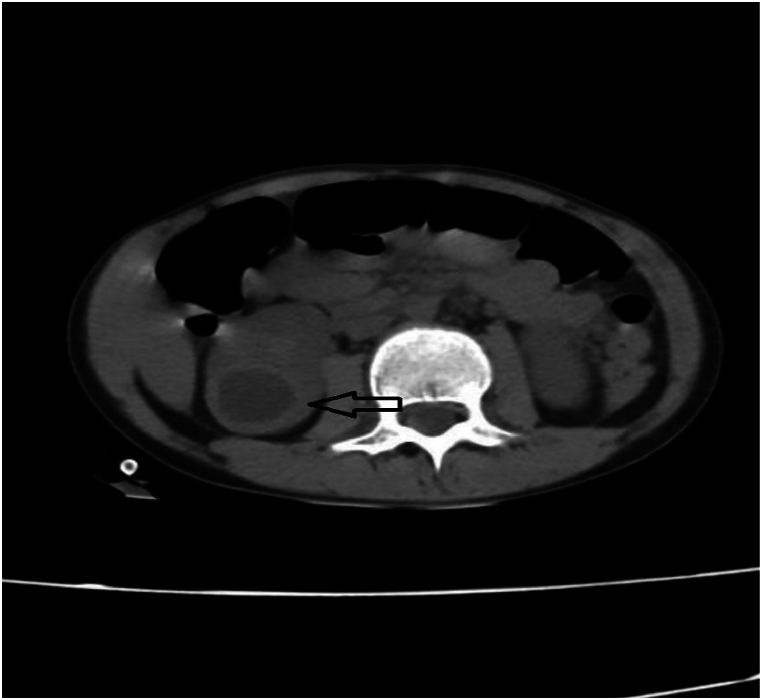
Kidney CT scan.

The attended case was a 10-year-old child who suffered shortness of breath and fever 20 days before the visit. She had gone to another hospital and was treated based on the diagnosis of empyema with a broad-spectrum antibiotic, and a chest tube was inserted. The analysis showed that the pleural fluid had zero white blood cells indicating the transudate and the culture was negative. Hydatid serology test Anti-Echinococcus ELISA (IgG) was analyzed, although the results were reported as unspecified. A lung CT scan was performed and moderate hydropneumothorax along with empyema in the right lung (right lower lobe) with mild mediastinal shift to the left was reported. The patient was referred to our hospital due to low clinical response with persistent fever and respiratory distress. During the examination, a decrease in breath sounds was heard on the right side. The initial chest X-ray of the patient has been demonstrated in Fig. 1. However, the lung CT scan was repeated indicating hydropneumothorax, fluid collection and consolidation at the right lower lob suspicious for abscess or empyema. Due to poor response to IV antibiotic therapy (150 mg/kg/day cefotaxim and 30 mg/kg/day clindamycin) and persistent fever, the patient underwent open surgery. During the surgery, a ruptured HC was seen in the right lung field, resulting in the removal of the cyst and its secretions by surgery, which was confirmed by the pathology report. In addition, the lung CT scan also showed parts of the kidney suspecting a renal hydatid cyst. As a result, a kidney CT scan (Fig. 2) was done and a multilocular cyst was seen in the lower bridge of the right kidney suspected to be a HC, even though she had no renal symptoms. Subsequently, the kidney cyst was completely removed by open surgery and the pathology was consistent with a HC. Subsequently, the patient was discharged with an oral albendazole prescription with a dose of 15mg/kg/day.

## 3 Discussion

HC is a zoonotic disease mostly affecting the liver and the lungs [1,3]. Additional organs affected by this disease include the musculoskeletal system, the kidneys and the brain [3]. Isolated renal HC is uncommon, and 44 % of individuals with this condition have other simultaneous diseases [9]. Typically, kidney involvement is unilateral in 85 % of cases [9]. Over time, the cyst enlarges within the kidney, commonly leading to a diagnosis around the age of 30 [10]. Nonetheless, renal HC can occasionally manifest at a younger age, as illustrated in this case. The most frequent symptom of renal HC is flank pain resulting from the pressure imposed by the cyst. An abdominal mass may indicate the presence of larger cysts [10,11]. Moreover, hydatid cysts are usually more common in the left kidney unlike the case presented above, where the right kidney had been affected. Thus, a study reported 10 cases of renal hydatid cysts, in which 8 of the cases the left kidney and 2 of them the right kidney was affected [12]. Another study noted 20 patients with renal hydatids, where 14 were affected on the left side and 6 on the right [13]. Additionally, a study on kidney HCs reported 18 patients, in which the affected kidney was 12 on the left and 6 on the right [14], which can be related to the shorter left renal artery.

In addition, imaging plays a crucial role in the diagnosis and staging of HC, although serology is less useful as it often yields false negative results [15]. Ultrasound stands out as the most critical diagnostic tool for hydatid disease, effectively identifying the presence of floating membranes, daughter cysts and hydatid sand typically found in these cystic lesions [16,17]. The treatment options for renal HC include medical therapy, percutaneous procedures, open surgery and minimally invasive techniques [18]. In treating renal HC, medical therapy should be used primarily before and after surgery, rather than as the main treatment strategy [11,18]. Moreover, the puncture-aspiration-injection-reaspiration (PAIR) technique is a form of percutaneous intervention, which is effective in about 70 % of cases and is specifically recommended for patients at high surgical risk with symptomatic HC [10]. In terms of surgical approaches, HC surgery can lead to anaphylactic shock and even death. To mitigate the risk of allergic reactions, it is advisable to carefully irrigate the cyst with a scolicidal solution (such as hypertonic sodium chloride, 0.5 % silver nitrate, 2 % formalin, 1 % iodine) before performing a cystectomy [9]. Additionally, in cases of isolated renal HC, kidney-sparing surgery should be prioritized [10,19]. In the illustrated case, the patient underwent a complete cystectomy while preserving the kidney tissue.

Pulmonary hydatid cyst (PHC) is endemic in many countries [[20], [21], [22]] where cattle breed and animals like pigs, goats, sheep, horses, camels and dogs are common [23]. In children, PHC is more common than liver cysts (64 % lung, 28 % liver) compared to adults (60 % liver, 30 % lung), as their lungs have higher elasticity [24](25). Likewise, intact PHC is frequently asymptomatic and it is sometimes accidently discovered on a chest radiograph [25]. However, intact cysts can also cause non-specific respiratory symptoms [26]. Subsequently, surgery is the gold standard in the treatment of PHC [27,28], as it aims to preserve the maximum lung tissue of children [27]. Therefore, the goal of the surgery should be to preserve lung function [9]. Conservative surgical techniques, such as cystectomy or cystotomy, constitute the choice surgical approach for intact cysts in every cyst size [[29], [30], [31]]. The PHC rupture might occur in the pleural space and it may cause pleural effusion, which is an important pulmonary complication in HC [32]. In the mentioned case, the cyst ruptured into the pleural cavity, which could be life-threatening. In addition, other complications are collapsed lung, simple pneumothorax, pleural thickening, and empyema [[32], [33], [34]]. Additional findings in the imaging include cavities with or without air-fluid levels, pneumonitis, and atelectasis in cases of ruptured lung cysts [35]. It should be noted that an air-fluid level is the most important radiological indication in patients with complicated cysts [33]. For complicated HC, surgical methods such as decortication, segmentectomy, thoracotomy, and lobectomy are more effective [32]. The case that has been discussed underwent a thoracotomy and decortication, and all cysts were successfully removed. Consequently, patients are exposed to various complications in surgery like, broncho-biliary fistula, empyema, unexpanded lung, pneumothorax, and spillage of hydatid contents. Additionally, no pulmonary function test was done while the patient was under treatment and after being cured. Thus, the patient mentioned underwent a six-month follow-up, in which no surgical complications were observed and there was no relapse, despite kidney and lung surgery.

## 4 Conclusion

In conclusion, in cases where pulmonary empyema does not respond to standard treatments, especially in endemic areas, hydatid cysts can be considered as another diagnosis. Even though it is rare, it is possible to find pulmonary and renal hydatid cysts together in children. In the mentioned case, surgery was proved to be safe for both cysts.

## CRediT authorship contribution statement

**Seyed Hossein Mirlohi:** Writing – review & editing, Resources, Project administration, Investigation, Data curation. **Sanaz Tajfirooz:** Writing – review & editing, Writing – original draft, Software, Methodology, Investigation, Formal analysis, Data curation. **Hojatollah Raji:** Investigation. **Sima Akhavan:** Data curation.

## Informed consent

The patient gave written informed consent to publish the current case report and any accompanying images. The editor-in-chief of this journal can review a copy of the written consent.

## Ethical approval

The Ethics Committee of Tehran University of Medical Sciences has approved this study. (code IR.TUMS.CHMC.REC.1402.177).

## Contributions

Each author declares substantial contributions through the following:

(1) The conception and design of the study, or acquisition of data, or analysis and interpretation of data [2], drafting the article or revising it critically for important intellectual content,

Please indicate for each author the author contributions in the text field below. Signatures are not required.

The conception and design of the study and analysis interpretation of data was done by S.H.M.

Drafting the article and revising it was done by S.T. and M.R.

## Approval of the submitted version of the manuscript

Please check this box to confirm that all co-authors have read and approved the version of the manuscript that is submitted. Signatures are not required.

## Declaration of competing interest

The authors declare that they have no known competing financial interests or personal relationships that could have appeared to influence the work reported in this paper.
